# Response of Demographic Rates of Tropical Trees to Light Availability: Can Position-Based Competition Indices Replace Information from Canopy Census Data?

**DOI:** 10.1371/journal.pone.0081787

**Published:** 2013-12-06

**Authors:** Steffi Grote, Richard Condit, Stephen Hubbell, Christian Wirth, Nadja Rüger

**Affiliations:** 1 Spezielle Botanik und Funktionelle Biodiversität, Universität Leipzig, Leipzig, Germany; 2 Center for Tropical Forest Science, Smithsonian Tropical Research Institute, Washington, District of Columbia, United States of America; 3 Department of Ecology and Evolutionary Biology, University of California Los Angeles, Los Angeles, California, United States of America; 4 German Centre for Integrative Biodiversity Research (iDiv) Halle-Jena-Leipzig, Leipzig, Germany; Beijing Forestry University, China

## Abstract

For trees in tropical forests, competition for light is thought to be a central process that offers opportunities for niche differentiation through light gradient partitioning. In previous studies, a canopy index based on three-dimensional canopy census data has been shown to be a good predictor of species-specific demographic rates across the entire tree community on Barro Colorado Island, Panama, and has allowed quantifying between-species variation in light response. However, almost all other forest census plots lack data on the canopy structure. Hence, this study aims at assessing whether position-based neighborhood competition indices can replace information from canopy census data and produce similar estimates of the interspecific variation of light responses. We used inventory data from the census plot at Barro Colorado Island and calculated neighborhood competition indices with varying relative effects of the size and distance of neighboring trees. Among these indices, we selected the one that was most strongly correlated with the canopy index. We then compared outcomes of hierarchical Bayesian models for species-specific recruitment and growth rates including either the canopy index or the selected neighborhood competition index as predictor. Mean posterior estimates of light response parameters were highly correlated between models (*r*>0.85) and indicated that most species regenerate and grow better in higher light. Both light estimation approaches consistently found that the interspecific variation of light response was larger for recruitment than for growth rates. However, the classification of species into different groups of light response, *e.g.* weaker than linear (decelerating) vs. stronger than linear (accelerating) differed between approaches. These results imply that while the classification into light response groups might be biased when using neighborhood competition indices, they may be useful for determining species rankings and between-species variation of light response and therefore enable large comparative studies between different forest census plots.

## Introduction

For trees in tropical forests, light is widely believed to be one of the most limiting resources and trade-offs resulting from differential responses of demographic rates (recruitment, growth, mortality) to light availability may contribute to the coexistence of the large number of tree species in these habitats [1]–[4]. However, quantifying how species' demographic rates change depending on light availability in hyper-diverse tropical forests is challenging and previous studies that analyzed the light response of trees in tropical habitats have yielded contradictory findings. Regarding recruitment, conclusions about species' light requirements range from high light dependence for around half of the 320 species in the tropical rainforest of La Selva, Costa Rica [5] to low light dependence for >80% of the analyzed 108 species on Barro Colorado Island (BCI), Panama [6]. However, these assessments were based on species' occurrences in gaps vs. non-gaps and high-canopy sites vs. low-canopy sites, respectively. Concerning growth, it has been proposed that reversals of growth rankings along the light gradient could promote species richness [7], [8]. But studies on tropical tree seedlings including few species (<35) also led to inconsistent results [9]–[12].

Recently, the degree of interspecific variation in light response has been quantified across an entire tropical tree community (>260 species) in a 50 ha forest census plot on Barro Colorado Island, Panama [13], [14]. These studies indicate that between-species variation in light response is much larger for recruitment than for growth. While light response of recruitment ranged from negative to strongly positive, growth rates of nearly all species increased less than linear with light and growth rankings in high and low light were highly correlated. Thus, the potential for niche differentiation through light gradient partitioning is thought to be larger for recruitment than for growth. Nevertheless, it was found that nearly all species regenerate and grow better in higher light and that for the majority of the species recruitment and growth rates increase less than linear with light availability [13], [14]. It is unclear whether these findings hold for other forests, or if the degree of interspecific variation in light response across a community varies with abiotic or biotic conditions. While abiotic factors, such as climate and soil composition, alter the availability of other resources (*e.g.* water and nutrients), biotic factors like tree density or canopy transparency additionally have a direct effect on the average and/or heterogeneity of light availability. Comparative studies covering a range of biotic and abiotic conditions are needed to answer these questions.

The greatest challenge of such comparative studies is estimating light availability. While information on demographic rates is collected by *e.g.* the Center for Tropical Forest Science (CTFS, http://www.ctfs.si.edu) or The Amazon Forest Inventory Network (RAINFOR, [15]) in long-term forest monitoring plots, measuring light availability at the top of thousands of individual trees in a heterogeneous forest canopy is extremely labor-intensive. For the studies at BCI, a canopy index was developed which is based on canopy census data that report presence vs. absence of vegetation in six height layers on a 5×5 m-grid in the census plot [13]. Until lidar data become widely available, this method is considered to be a valuable trade-off between time-consuming direct measurements and a coarse classification of light availability into few categories, *e.g.* gap/non gap [16], [17]. However, this approach is not applicable in most forests because few forest plots include high-resolution measures of canopy structure.

An alternative approach to estimating light availability is using neighborhood competition indices (NCIs) that are based on information on the size and distance of neighboring trees. Since diameter at breast height (dbh) and position are among the most frequently measured properties in forest census plots [15], [18], neighborhood competition indices enable comparisons among a large number of forest plots. The dbh can be used as a proxy for tree height [17], [19], which in turn can be used as a proxy for canopy size and shading [20]. And there is a long tradition of neighborhood analyses that are based on the dbh and position of the neighboring trees (e.g. [21]–[23]). However, we assume that a canopy index based on three-dimensional censuses of canopy density is more refined than neighborhood competition indices because the latter cannot account for asymmetric crowns or leaning trees.

The objectives of this study are (1) to explore to which degree information from canopy census data can be replaced by neighborhood competition indices and (2) to investigate whether recruitment and growth models using neighborhood competition indices instead of the canopy index reveal similar patterns of interspecific variation in light response. Using either the canopy index or a neighborhood competition index as proxy for light availability, we compared outcomes of hierarchical models for recruit numbers and tree growth for >250 tree species on BCI. Species-specific parameters of these models were estimated in a Bayesian framework to account for different sources of uncertainty and to correctly weight data from rare species [24], [25]. If the coefficients of the canopy index and neighborhood competition indices were highly correlated, the neighborhood competition indices could be used to determine species rankings and be applied in comparative analyses among census plots. If estimates of species' light response of both approaches would additionally fall on a 1∶1 line, neighborhood competition indices could replace canopy census data without a substantial loss of information.

## Methods

### Study site

We used data from the BCI Forest Dynamics Plot, which was established in lowland moist forest on Barro Colorado Island, a former hilltop in the Panama Canal (9°9′N, 79°51′W). In 1980, a 50 ha rectangular permanent plot (48 ha of old-growth forest and 2 ha of secondary forest) was laid out on the island [26]. Elevation of the plot is 120–155 m a.s.l., the average annual temperature and rainfall are 27°C and 2600 mm, respectively. Rainfall is seasonal with a rainy season from April to November and a dry season starting in December [27], [28]. For a detailed description of the site see [27]. Barro Colorado Island is managed exclusively for field research by the Smithsonian Tropical Research Institute (STRI), which has been granted long-term custodianship over the island by Panama's Environmental Authority.

### Recruitment and growth data

On BCI, forest censuses were conducted in 1982, 1985 and every five years thereafter (http://www.ctfs.si.edu, [18]). Census data include stem diameter at breast height (dbh), xy-coordinates and species identity of all freestanding woody plants with a dbh of at least 1 cm. In this study, we used the census intervals 1985–1990 and 1990–1995, because these are the only two census intervals with consistent canopy census data for estimating light availability [14]. To avoid edge effects, we imposed a 30 m margin along each side of the plot and restricted the analysis to the 940×440 m core region.

We defined recruits as all individuals that were absent in the first but present in the second census, *i.e.* all trees that had passed the threshold of 1 cm dbh during this period. This yielded a total of 31,152 recruits of 253 species in the first, and 16,856 recruits of 231 species in the second census interval. We divided the census plot into 5×5 m grid cells and determined the number of recruits for each grid cell and each species.

Growth rates were defined as the dbh increment of a stem between two censuses divided by the time elapsed (mm/yr). For trees with multiple stems, we used only the single largest stem and we excluded cases where a tree survived but its stem was measured at a different position, or where one stem broke so a resprouted stem of the same tree was measured; palms were excluded due to lack of secondary growth. This resulted in growth records for 135,788 trees of 264 species in 1985–1990 and 139,625 trees of 268 species in 1990–1995. Because dbh was rounded down to the nearest 5 mm for all stems <55 mm in 1985 but not in 1990, it was necessary to round 1990 dbh values below 55 mm down as well before calculating growth rates over 1985–1990; no rounding was done after 1985 [14]. Rounding down may bias growth estimates of small stems, but [29] showed that this bias is minimal.

### Canopy index

Canopy index values (CAI) were taken from Rüger *et al.* [13], [30] and represent the average of yearly estimates over one census interval (1985–1990 or 1990–1995). The calculation of the canopy index relies on canopy census data which reported presence vs. absence of vegetation in six height intervals (0–2, 2–5, 5–10, 10–20, 20–30 and ≥30 m) every 5 m across the plot. These data were used to calculate a shade index for any focal point in the plot as a weighted sum of vegetation above the point and <20 m away. The shade index was then converted into an estimate of light availability by matching the distribution of shade estimates at 2 m height with a published distribution of irradiance at 1 m height that was based on 396 direct measurements on a nearby site on BCI in 1993 [31]. For details see [13], [30]. For recruitment models, the canopy index was calculated for the center of each 5×5 m grid cell at 2 m height, because each recruit must have surpassed that height during the recruitment census interval. Given that the canopy census was performed with a resolution of 5×5 m, we assume that there is little variation in estimated light availability within grid cells. For growth models, the canopy index was calculated for the top of the crown of each individual tree. The height of the tree was estimated as a function of its dbh [30].

### Neighborhood competition indices

To approximate the competition for light by neighboring trees, we used distance-dependent neighborhood competition indices (NCIs). These indices are based upon the traditional assumption that the competitive pressure a tree experiences can be described as a function of the size and distance of neighboring trees (*e.g.* [21], [22], [32]). We used the equation 
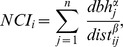
where *dbh_j_* is the dbh in cm of one of *n* neighboring trees within a 30 m radius and *dist_ij_* the distance to the focal point *i* in m, while *α* and *β* are parameters which describe the weighting of tree size and distance, respectively [33]. For grid cells, the neighborhood competition index was calculated for the center of the 5×5 m grid cell, whereas for individual trees their discrete position was used. Since we were interested in competition for light, we considered only trees taller than the focal tree as neighbors. We systematically varied *α* and *β* from 0 to 3 in steps of 0.2, which resulted in 256 different neighborhood competition index values for a given focal point. This is a rather large range given that most neighborhood competition indices use values of *α* between 1 and 2 and values of *β* between 0 and 1 [21], [32], [34]–[37] and that *α* = 3 or *β* = 3 result in eight-fold increase of the shading effect when the dbh is doubled and an eight-fold decrease of the shading effect when the distance to the focal tree is doubled, respectively. In contrast to the canopy index which integrates information on the canopy structure over five years by averaging shade estimates from yearly canopy censuses [13], [30], the neighborhood competition indices are calculated from census data in the first year of the forest census interval (1985 or 1990).

The canopy index was lognormally distributed, while the neighborhood competition indices were normally distributed, so we log-transformed the canopy index but not the neighborhood competition indices. For grid cells (recruitment) and individual trees (growth), we selected the neighborhood competition index, *i.e.* the combination of *α* and *β*, which had the highest correlation (Pearson's *r*) with the log(canopy index) across both census intervals to model recruit numbers and growth rates in subsequent analyses. Our goal was to compare models of demographic rates with either the canopy index or the selected neighborhood competition index as predictor and to evaluate how close parameters of alternative models are to a 1∶1 relationship. Therefore, we converted the neighborhood competition index into the log(canopy index) to obtain a similar range of light estimation values for the models to allow a direct comparison of parameters.

The conversion was done using quantile regression to approximate the median of the log(canopy index) at a given neighborhood competition index rather than the mean, because this method is less sensitive to outliers. Since the relationship between log(canopy index) and the neighborhood competition index was linear for grid cells (recruitment), but non-linear for trees (growth), we included an additional quadratic term into the regression for trees: 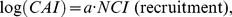


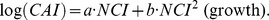



The intercept was forced to be zero because the absence of taller trees in the neighborhood (neighborhood competition index  = 0) should refer to 100% irradiance (canopy index  = 1, log(canopy index)  = 0). We fitted interval-specific regressions as well as a general regression pooling data of both census intervals. The coefficients of the regressions were used to determine the interval-specific transformed neighborhood competition index (NCI_ts_) and the general transformed neighborhood competition index for both intervals (NCI_tg_). For comparison, we also ran models including raw untransformed neighborhood competition index values.

### Recruitment model

In modeling light response of recruit numbers in 5×5 m grid cells, we followed [13]. We used a power function (linear log-log relationship) to describe the relationship between the predicted number of recruits of species *j* in grid cell *i* (*pred_ij_*) and light:
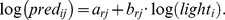



To compare the two indices, the term ‘log(*light_i_*)’ represents either the log(canopy index), the transformed neighborhood competition indices (NCI_ts_ or NCI_tg_) or the untransformed neighborhood competition index. All predictors were centered. The parameter *b_rj_* describes the species-specific light response of recruitment, and since the equation above is equivalent to
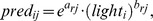

*b_rj_*<0 indicates a negative response of recruitment to light, whereas 0<*b_rj_*<1, *b_rj_* = 1 and *b_rj_*>1 indicate a decelerating, linear or accelerating response to light, respectively. This interpretation of *b_rj_* only holds if the term ‘log(*light_i_*)’ refers to the log(canopy index) or the neighborhood competition indices that are transformed into the log(canopy index), but not for the untransformed neighborhood competition index.

As there are other factors, apart from light, that lead to a spatial clustering of recruit numbers, such as the distribution of seed trees, heterogeneous seed dispersal or heterogeneous soil conditions [38]–[41], we used a negative binomial distribution with the species-specific clumping parameter *k_j_* to account for overdispersion of recruit numbers:
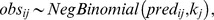
where *obs_ij_* is the observed number of recruits of species *j* in grid cell *i*.

As Rüger *et al.* [13] showed that both *a_r_* and *b_r_* may vary in a systematic way with the abundance of a species, we modeled species parameters as a function of abundance (*abun*), *i.e.* the number of individuals of a species in the plot at the beginning of a census interval: 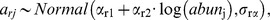


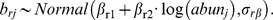



The standard deviations *σ_rα_* and *σ_rβ_* describe the interspecific variation of the parameters *a_rj_* and *b_rj_* at a given abundance. As we did not have prior knowledge of the parameter *k_j_* and the hyperparameters, we used uninformative flat priors:
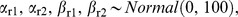


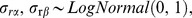


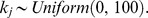



We chose an upper bound of 100 for *k_j_*, because for *k_j_*>10, the negative binomial already approximates a spatially homogeneous Poisson distribution [42].

We computed the posterior distributions of the parameters and hyperparameters with a Markov chain Monte Carlo method that it is a hybrid of the Metropolis Hastings algorithm and the Gibbs sampler [25], [43]. The step size was adjusted during the burn-in phase in such a way that acceptance rate was kept around 0.25 [43]. We ran two chains with different initial values and monitored convergence with Gelman and Rubin's convergence diagnostics where values <1.1 indicate that the chains have converged well [44]. We used a burn-in period of 1000 steps and a sampling period of 6000 steps, because parameters and hyperparameters only needed 500 to 800 iterations to converge. All analyses were performed using the Software package R version 2.15.1 [45]. The R code for the hierarchical Bayesian model for recruitment can be found in Appendix S1.

### Growth model

In modeling growth, we followed [14]. We fit a two-level hierarchical model in which individual tree growth was a species-specific function of light availability and initial dbh, and species-level parameters were predicted by the species' abundance. At the core of the model is the functional relationship predicting the absolute dbh growth rate (mm/yr) of individual *i* of species *j* (*pred_ij_*) as a power function of light availability and initial dbh,
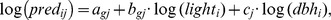
where parameters *a_gj_*, *b_gj_* and *c_j_* describe the intrinsic growth rate and the light and size response of growth of species *j*, respectively [14]. Again, to compare the two indices, the term ‘log(*light_i_*)’ either represents the centered log(canopy index), the transformed and centered neighborhood competition indices (NCI_ts_ or NCI_tg_) or the untransformed and centered neighborhood competition index. The dbh was centered on 50 mm dbh. As in the model for recruitment, the parameter *b_gj_* describes the species-specific response of growth to light availability from negative to decelerating, linear and accelerating increase, represented by *b_gj_*<0, 0<*b_gj_*<1, *b_gj_* = 1 and *b_gj_*>1, respectively. We included the dbh into the model because tree size significantly affects tree growth in many species [14].

Process error, *i.e.* variation of growth at a given light availability and dbh, was modeled using a lognormal distribution 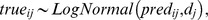
where *true_ij_* is the estimated true growth rate of tree *i*. The process error (*d_j_*) was estimated for each species. Using a lognormal distribution, the process error automatically scales with predicted growth. The process error (*d_j_*) was assumed to vary lognormally across the community with hyperparameters *δ_1_* and *δ_2_*:



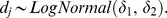



Data entered our model as the observed annual dbh growth of individual *i* of species *j* (*obs_ij_*, mm/yr) and were assumed to be subject to measurement error. We used previous estimates of two types of measurement error: Routine error caused by a slightly different placement of the callipers or tape measure, and large error caused by missing a decimal place or recording a number with the wrong tree. These were modeled as the sum of two normal distributions [14], [46]. Thus,
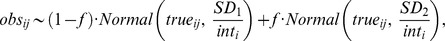
with *SD_1_* describing the size-dependent error component and *SD_2_* the size-independent error component affecting *f* = 2.7% of the observations. Standard deviations have to be adjusted to the time period elapsed between the two dbh measurements of the tree (*int_i_*) from which the annual growth rate has been calculated.

As for recruitment, species' parameters were modeled as a function of a species' abundance: 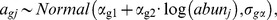


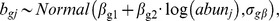


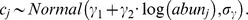



As we did not have prior knowledge about the distribution of the hyperparameters, we used uninformative flat priors:
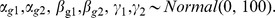



The posterior distributions of all parameters were computed using “Filzbach”, a software library for performing Metropolis Hastings Markov chain Monte Carlo parameter estimations (http://research.microsoft.com/en-us/projects/filzbach/). We used Filzbach because the growth model required a much longer computation time than the recruitment model, and Filzbach runs much faster than R. As we did not define priors for *σ_gα_*, *σ_gβ_*, *σ_γ_*, *δ_1_* and *δ_2_*, Filzbach automatically imposed an uninformative prior distribution for these parameter values. The code can be found in Appendix S2.

In contrast to the parameter estimation for the recruitment model where in each iteration all parameters were updated sequentially according to the conditional probability distribution, posterior distributions of the parameters of the growth model were sampled from the joint probability distribution, and in each step only one parameter was updated. We ran the model with a burn-in phase of 3 million iterations and a sampling phase of additional 3 million steps. Due to the long computation times, we ran only one chain with each of the indices (transformed neighborhood competition indices (interval-specific and general), untransformed neighborhood competition index and canopy index) and convergence was assessed by visual inspection.

### Analysis of results

We computed the mean and the 95% credible intervals (CI) of the posterior distributions of all parameters in both models, with the different neighborhood competition indices and the canopy index for both intervals. We compared the distribution of *b_r_* and *b_g_* across the community when using the different indices in the models. For models with the log(canopy index) or the transformed neighborhood competition indices (NCI_ts_ and NCI_tg_) as proxy for light, species were grouped into different classes of light response (*i.e.* negative, decelerating, accelerating) based on the mean posterior estimate of *b_r_* and *b_g_* as well as based on the 95% CI (*i.e.* the entire 95% CI had to be <0, between 0 and 1, or >1), and we determined the consistency of those classifications across census intervals and light estimation approaches.

## Results

### Recruitment

**Figure 1:**
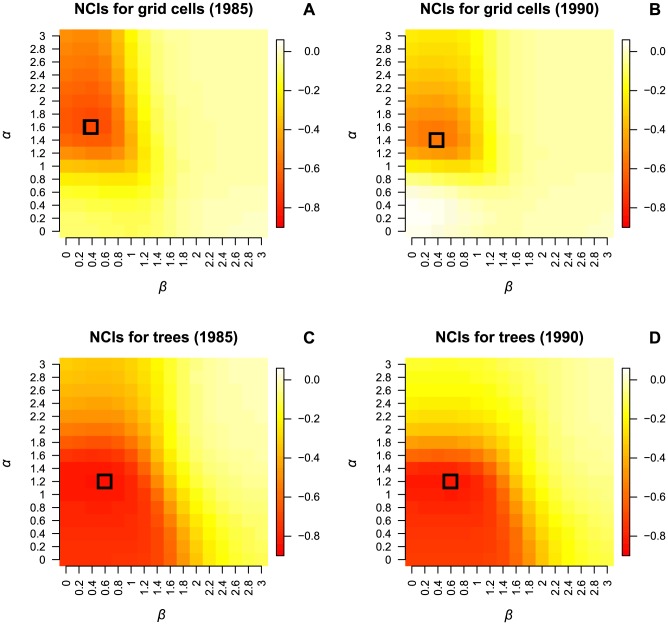
Correlation between log(canopy index) and neighborhood competition indices with different values of *α* and *β*. The correlation is based on the canopy index and neighborhood competition indices (NCIs) for 16,544 5×5 m grid cells (A, B) and for 135,788 (C) and 139,625 trees (D) in the BCI forest plot. For grid cells, the highest correlation was achieved with *α* = 1.6 and *β* = 0.4 in the first, and *α* = 1.4 and *β* = 0.4 in the second census (highlighted). For individual trees, the highest correlation was achieved with *α* = 1.2 and *β* = 0.6 (highlighted) in both censuses. Colors indicate Pearson's *r*.

**Figure 2:**
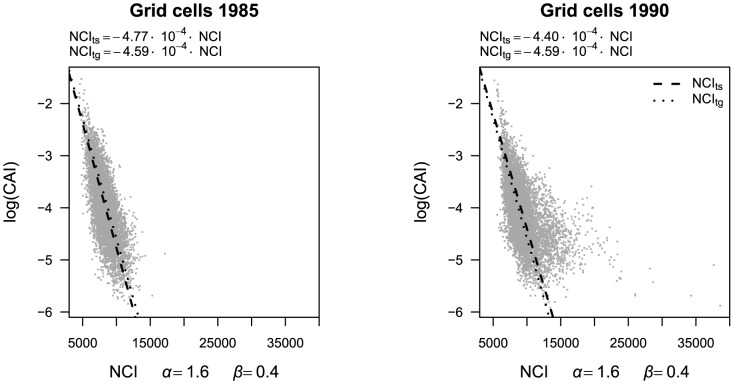
Best neighborhood competition index (NCI, *α* = 1.6, *β* = 0.4) versus canopy index (CAI). Lines show quantile regressions to convert neighborhood competition index (NCI) into an interval-specific estimate of log(canopy index) (NCI_ts_, dashed line) or into a general estimate of log(canopy index) which is based on pooled data from both census intervals (NCI_tg_, dotted line).

For grid cells, the correlation between the log(canopy index) and the neighborhood competition indices was negative in almost all cases and ranged from 0.004 to −0.69 (Figure 1A, B). Generally, the canopy index was best predicted with *α*>1 and *β*<1. The correlation was strongest for *α* = 1.6 and *β* = 0.4 in the first, and *α* = 1.4 and *β* = 0.4 in the second census interval with *r* = −0.69 and *r* = −0.56, respectively. As our aim was to identify a single combination of *α* and *β* to conduct subsequent analyses, we pooled data from both intervals and identified *α* = 1.6 and *β* = 0.4 as the overall ‘best’ combination with only a minor loss of correlation for the second census interval (*r* = −0.55). Quantile regressions converting the neighborhood competition index into log(canopy index) tended to underestimate high light conditions and overestimate low light conditions (Figure 2). Nevertheless, the range of transformed neighborhood competition index values was wider than the range of the log(canopy index), because especially in the second census interval some outliers with very high neighborhood competition index values were converted to extremely low log(canopy index) values (Figure 2).

**Figure 3:**
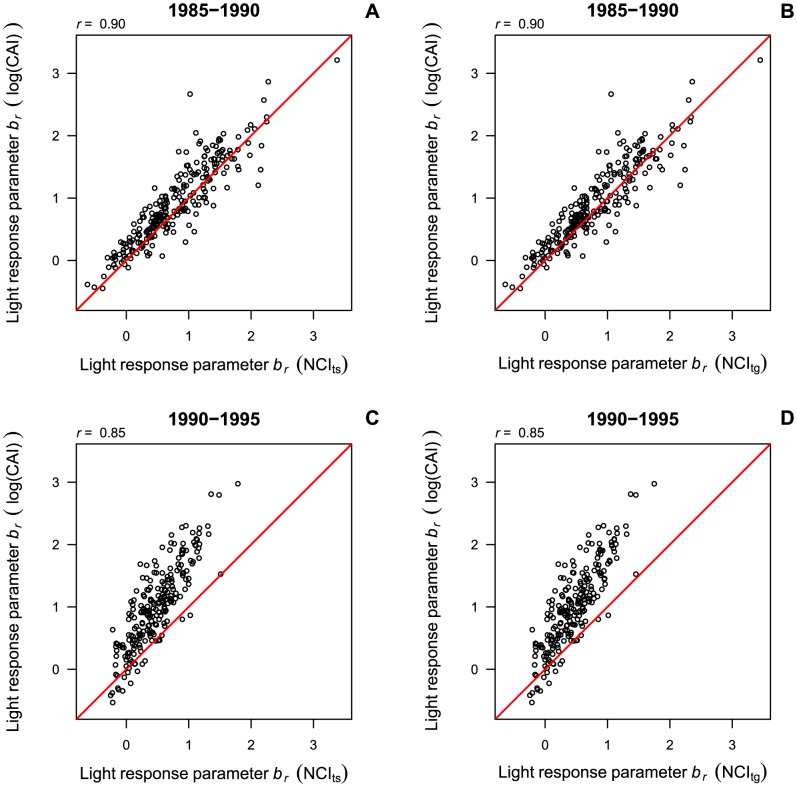
Species-specific light response of recruitment (*b_r_*) in models with neighborhood competition index versus canopy index. Panels show posterior means of *b_r_* in models with the interval-specific neighborhood competition index (NCI_ts_, A, C) and the general neighborhood competition index (NCI_tg_, B, D) compared to *b_r_* in models with the canopy index (log(CAI)). The correlation between the coefficients (*r*) and the 1∶1 line are indicated.

Light response parameters *b_r_* were highly correlated between models including the canopy index or the transformed neighborhood competition indices (*r*>0.85, Figure 3). When using raw (untransformed) neighborhood competition index values, the correlations of the light response parameters *b_r_* were practically the same as for converted neighborhood competition index values (*r* = 0.90 in the first and *r* = 0.85 in the second census interval, Appendix S3). While the parameter estimates of the transformed neighborhood competition indices and the canopy index were relatively similar in the first census interval, there was considerable deviation from a 1∶1 relationship in the second census interval, where *b_r_* was underestimated by the neighborhood competition indices, especially for light-demanding species.

**Figure 4:**
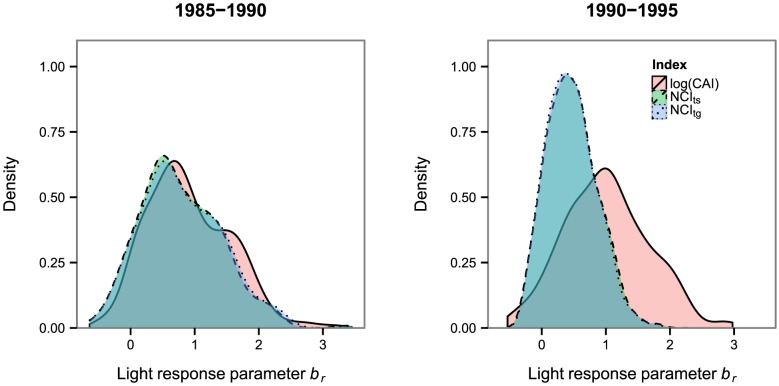
Community-wide distribution of the light response of recruitment (*b_r_*). Light is estimated either with the canopy index (log(CAI)), the interval-specific neighbordood competition index (NCI_ts)_ or the general neighborhood competition index (NCI_tg_). The underlying histogram can lead to sharp edges of the density plot.

The distribution of the posterior means of *b_r_* across the community revealed that the majority of the species regenerated better in higher light (*b_r_*>0, Figure 4). This result was independent of the light estimation approach. In the first census interval, the distributions of *b_r_* were similar across all light estimation approaches. However, in the second census interval, the range of between-species variation in light response was underestimated by the neighborhood competition indices compared to the canopy index since higher values of *b_r_* were underestimated by the neighborhood competition indices.

Although all light estimation approaches revealed that the majority of species responded to light in a decelerating manner, there were differences in classifying light responses into negative (*b_r_*<0), decelerating (0<*b_r_*<1), and accelerating (*b_r_*>1) responses (Table 1). The neighborhood competition indices identified more species with a negative or decelerating response to light and fewer species with an accelerating response to light than the canopy index. Additionally, using the canopy index led to more classifications that were consistent over the two census intervals.

**Table 1:** Classification of tree species on the basis of their recruitment responses to light availability: negative (*b_r_*<0), decelerating (0<*b_r_*<1) and accelerating (*b_r_*>1).

Light estimate	Census interval	Light response of recruitment							
		defined by posterior mean of *b_r_*				defined by 95% CI of *b_r_*			
		negative	decelerating	accelerating	inconsistent	negative	decelerating	accelerating	inconsistent
log(CAI)	1985–1990	11	141	101	–	2	33	32	186
log(CAI)	1990–1995	12	107	112	–	2	24	30	175
log(CAI)	both	5	92	72	51	1	15	21	183
NCI_ts_	1985–1990	24	143	86	–	5	39	19	190
NCI_ts_	1990–1995	22	186	23	–	5	29	2	195
NCI_ts_	both	10	113	15	82	4	17	2	197
NCI_tg_	1985–1990	24	139	90	–	5	37	22	189
NCI_tg_	1990–1995	23	186	22	–	5	30	2	194
NCI_tg_	both	10	109	14	87	4	15	2	199
NCI_ts_ + log(CAI)	both	3	70	14	133	1	7	2	210
NCI_tg_ + log(CAI)	both	3	67	13	137	1	7	2	210

CAI: canopy index, NCI_ts_: interval-specific transformed neighborhood competition index, NCI_tg_: general transformed neighborhood competition index. Classifications for both intervals refer to the number of species that had consistent classification over both intervals. The last two lines indicate the number of species that were classified into the same group by the log(canopy index) and a neighborhood competition index in both intervals.

Finally, consistent classifications through neighborhood competition indices and the canopy index and over two intervals were made for around 40% of the number of species that had recruits in both census intervals (n = 220): 3 negative responses, 70 (NCI_ts_) and 67 (NCI_tg_) decelerating responses and 14 (NCI_ts_) and 13 (NCI_tg_) accelerating responses. When classifications of light responses were based on the 95% CI instead of the posterior mean estimate, only between 36 and 56 species could be classified with the different indices in a single census (Table 1). Consistent classifications across two census intervals were even rarer.

All light response parameters were significantly negatively correlated with abundance, indicating that light-demanding species tend to be rare and that abundant species tend to respond less to light (Table 2).

**Table 2:** Posterior estimates of hyperparameters for the species-specific parameters of the recruitment model.

Parameter	Light estimate	Intercept		Slope (abundance)		Standard deviation	
		(*α_r1_*, *β_r1_*)		(*α_r2_*, *β_r2_*)		(*σ_rα_*, *σ_rβ_*)	
		1985–1990	1990–1995	1985–1990	1990–1995	1985–1990	1990–1995
Intercept (*a_r_*)	log(CAI)	−10.59	−11.34	0.78	0.81	0.97	0.88
Intercept (*a_r_*)	NCI_ts_	−10.53	−10.96	0.78	0.77	0.97	0.92
Intercept (*a_r_*)	NCI_tg_	−10.50	−10.97	0.77	0.77	0.97	0.92
Light response (*b_r_*)	log(CAI)	1.60	1.99	−0.15	−0.20	0.67	0.63
Light response (*b_r_*)	NCI_ts_	1.54	1.06	−0.16	−0.12	0.63	0.39
Light response (*b_r_*)	NCI_tg_	1.59	1.05	−0.17	−0.12	0.65	0.37

CAI: canopy index, NCI_ts_: interval-specific transformed neighborhood competition index, NCI_tg_: general transformed neighborhood competition index. Models were run with different light estimates and for two census intervals. All hyperparameters were significantly different from 0 (based on 95% CIs).

### Growth

**Figure 5:**
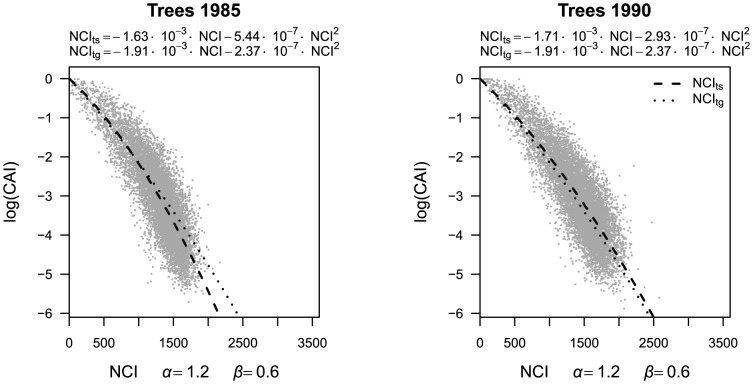
Best neighborhood competition index (NCI, *α* = 1.2, *β = *0.6) versus canopy index (CAI). Lines show quantile regressions to convert neighborhood competition index (NCI) into an interval-specific estimate of log(canopy index) (NCI_ts_, dashed line) or into a general estimate of log(canopy index) which is based on pooled data from both census intervals (NCI_tg_, dotted line).

For individual trees, the correlation between the log(canopy index) and the neighborhood competition indices was negative in all cases and ranged from −0.01 to −0.84 (Figure 1C, D). Values of *α* and *β*<2 were best to approximate the canopy index and the combination *α* = 1.2 and *β* = 0.6 yielded the highest correlation in both census intervals (*r* = −0.84). Quantile regressions converting the neighborhood competition index into log(canopy index) tended to underestimate high light conditions and overestimate low light conditions (Figure 5). Interval-specific and general regressions were only slightly different, especially in the second census interval.

**Figure 6:**
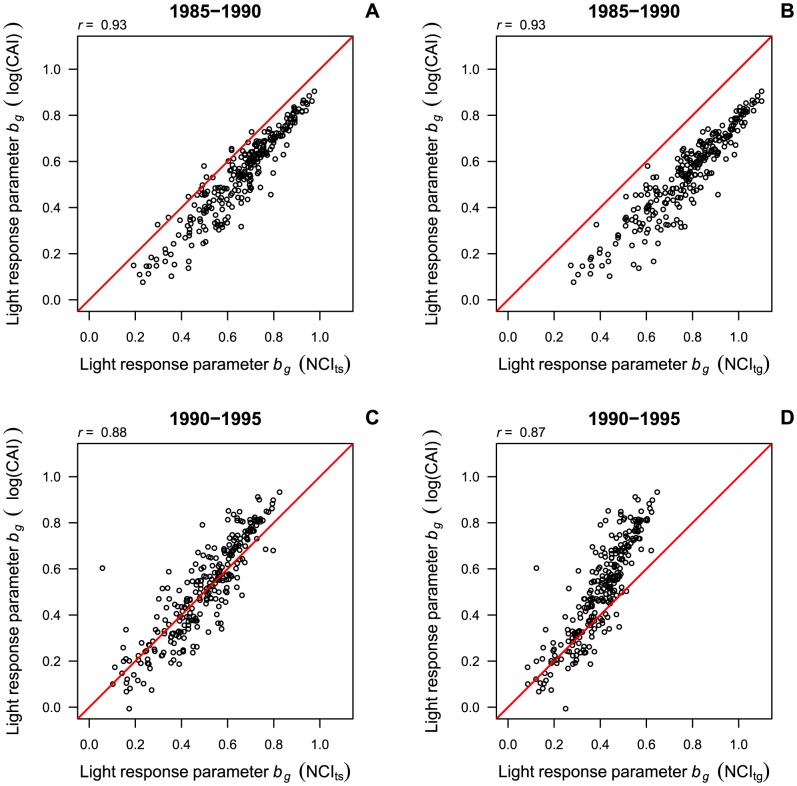
Species-specific light response of growth (*b_g_*) in models with neighborhood competition index versus canopy index. Panels show posterior means of *b_g_* in models with the interval-specific neighborhood competition index (NCI_ts_, A, C) and the general neighborhood competition index (NCI_tg_, B, D) compared to *b_g_* in models with the canopy index (log(CAI)). The correlation between the coefficients (*r*) and the 1∶1 line are indicated.

Similar to recruitment, the correlation of the posterior means of *b_g_* between models of growth rates with the canopy index and models with neighborhood competition indices was high (*r*>0.87, Figure 6). When using raw (untransformed) neighborhood competition index values, the correlations of the light response parameters *b_g_* were slightly lower than those obtained with transformed neighborhood competition index values (*r* = 0.90 in the first and *r* = 0.85 in the second census interval, Appendix S3). For the transformed neighborhood competition indices the deviation from a 1∶1 relationship showed a different pattern than in recruitment models. For 1985–1990, values of *b_g_* were overestimated by the neighborhood competition indices, especially by the general transformed neighborhood competition index. For 1990–1995, in turn, higher values of *b_g_* tended to be underestimated by the general transformed neighborhood competition index compared to the canopy index, while parameter estimates with the interval-specific transformed neighborhood competition index and the canopy index were scattered quite evenly around the 1∶1 line (Figure 6).

**Figure 7:**
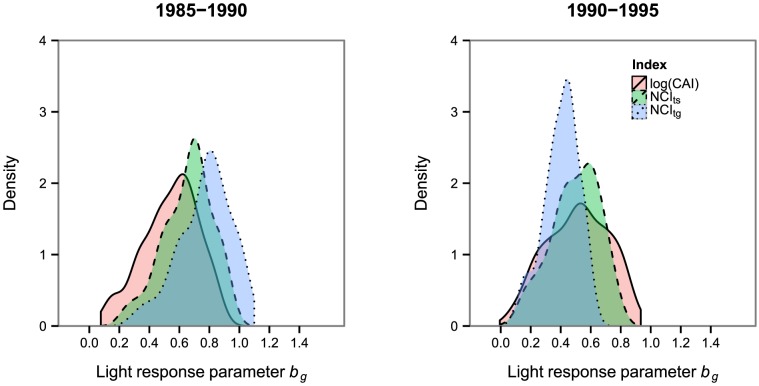
Community-wide distribution of the light response of growth (*b_g_*). Light is estimated either with the canopy index (log(CAI)), the interval-specific neighbordood competition index (NCI_ts)_ or the general neighborhood competition index (NCI_tg)_. The underlying histogram can lead to sharp edges of the density plot.

Compared to recruitment, responses of growth rates to light availability showed a less variable pattern of light responses across the community (Table 3, Figure 7). Posterior means of *b_g_* fell between 0 and 1 for almost all species and light estimation approaches. Only 24 species were identified to have an accelerating response to light (general transformed neighborhood competition index in the first census interval) and only one species, *Piper cordulatum* (canopy index), had a negative value of *b_g_* in the second census interval. Therefore, almost all species were classified with a positive but decelerating response to light, even when classifications were based on 95% CIs.

**Table 3:** Classification of tree species on the basis of their growth responses to light availability: negative (*b_g_*<0), decelerating (0<*b_g_*<1) and accelerating (*b_g_*>1).

Light estimate	Census interval	Light response of recruitment							
		defined by posterior mean of *b_g_*				defined by 95% CI of *b_g_*			
		negative	decelerating	accelerating	inconsistent	negative	decelerating	accelerating	inconsistent
log(CAI)	1985–1990	0	264	0	–	0	210	0	54
log(CAI)	1990–1995	1	267	0	–	0	191	0	77
log(CAI)	both	0	258	0	1	0	183	0	76
NCI_ts_	1985–1990	0	264	0	–	0	179	0	85
NCI_ts_	1990–1995	0	268	0	–	0	225	0	43
NCI_ts_	both	0	259	0	0	0	175	0	84
NCI_tg_	1985–1990	0	240	24	–	0	96	0	168
NCI_tg_	1990–1995	0	268	0	–	0	266	0	2
NCI_tg_	both	0	239	0	20	0	94	0	165
NCI_ts_ + log(CAI)	both	0	258	0	1	0	157	0	102
NCI_tg_ + log(CAI)	both	0	238	0	21	0	85	0	174

CAI: canopy index, NCI_ts_: interval-specific transformed neighborhood competition index, NCI_tg_: general transformed neighborhood competition index. Classifications for both intervals refer to the number of species that had consistent classification over both intervals. The last two lines indicate the number of species that were classified into the same group by the log(canopy index) and a neighborhood competition index in both intervals.

As for recruitment, but to a lesser extent, light response of growth rates (*b_g_*) was significantly negatively correlated with abundance, indicating that light-demanding species tend to be less common and that abundant species tend to respond less to light (Table 4).

**Table 4:** Posterior estimates of hyperparameters for the species-specific parameters of the growth model.

Parameter	Light estimate	Intercept		Slope (abundance)		Standard deviation	
		(*α_g1_*, *β_g1_*, *γ_1_*)		(*α_g2_*, *β_g2_*, *γ_2_*)		(*σ_gα_*, *σ_gβ_*, *σ_γ_*)	
		1985–1990	1990–1995	1985–1990	1990–1995	1985–1990	1990–1995
Intercept (*a_g_*)	log(CAI)	−0.06	−0.19	−0.06	−0.08	0.48	0.48
Intercept (*a_g_*)	NCI_ts_	0.01	−0.11	−0.07	−0.09	0.49	0.54
Intercept (*a_g_*)	NCI_tg_	−0.27	−0.03	−0.06	−0.09	0.49	0.55
Light response (*b_g_*)	log(CAI)	0.86	0.83	−0.07	−0.07	0.16	0.20
Light response (*b_g_*)	NCI_ts_	0.95	0.75	−0.06	−0.06	0.15	0.17
Light response (*b_g_*)	NCI_tg_	1.07	0.60	−0.07	−0.04	0.17	0.12
Size effect (*c_g_*)	log(CAI)	−0.21	−0.09	0.04	0.03	0.26	0.26
Size effect (*c_g_*)	NCI_ts_	−0.30	−0.04	0.04	0.02	0.24	0.24
Size effect (*c_g_*)	NCI_tg_	−0.27	−0.01	0.03	0.02	0.23	0.23

CAI: canopy index, NCI_ts_: interval-specific transformed neighborhood competition index, NCI_tg_: general transformed neighborhood competition index. Models were run with different light estimates and for two census intervals. All hyperparameters were significantly different from 0 (based on 95% CIs).

## Discussion

In this study we assessed the ability of neighborhood competition indices to replace information from canopy census data. We compared recruitment and growth models that included as predictors either a canopy index, which was derived from canopy census data or a neighborhood competition index that was most strongly correlated with the canopy index. Comparison of parameter estimates indicated that species-specific light response parameters of alternative models were tightly correlated, but that there was considerable deviation from a 1∶1 relation. That is, a given species would be assigned a similar rank in the community concerning the strength of its light response, but the specific value for that light response parameter would generally be different in canopy index and neighborhood competition index models. Hence, using neighborhood competition indices to approximate competition for light seems to be a simple and robust method when the aim of the study is to compare species rankings or between-species variation of light responses among different forests.

### Relationship between canopy index and neighborhood competition indices

The parameters of the neighborhood competition index that led to the highest correlation with the canopy index were *α* = 1.6 and *β* = 0.4 for recruits and *α* = 1.2 and *β* = 0.6 for individual trees. These optimal combinations performed well in both census intervals and indicate that for small trees (recruits) the size of neighbors is more important for light availability and that competitive effects decrease less with distance to the focal tree as compared to larger trees. To resemble the canopy index, our optimal values of *α* were between 1 and 2 and thus, between a linear increase of shading with the dbh (*α* = 1) and a linear increase of shading with the basal area of neighboring trees (*α* = 2). Since the canopy area also increases less than linearly with the basal area (the canopy diameter increases less than linearly with the dbh, *e.g.* [47]), we hypothesize that shading is related to canopy area. Previously, neighborhood competition indices have not been used to describe explicitly the light conditions, but competition in general, including below-ground competition and competition for space [33]. Competitive effects in tropical forests are often assumed to scale with the basal area of neighboring trees [34]–[36], thus the relationship between size and competitive effect is assumed to be steeper than the relationship between size and shading we found here.

Regarding the decrease of shading intensity with the distance of neighboring trees, values of *β* were between 0 and 1. While *β* = 0 results in no decrease of the shading effect of neighbors with distance, for *β* = 1 the shading effect is divided by the distance to the neighboring tree, *i.e.* the effect is halved when the distance is doubled. No decrease of overall competition with distance was assumed by *e.g.* Gourlet-Fleury & Houllier [37] for neighbors within 30 m from the focal tree in a tropical forest, whereas *e.g.* Hegyi's index [21] divides the competitive effect of neighbors in jack-pine stands by the distance.

A study that was conducted in a Puerto Rican forest plot made use of the same neighborhood competition index equation, but estimated *α* and *β* individually for 11 dominant target tree species [48]. In that study the neighborhood competition indices were optimized for predicting growth rates, and species-specific optimal values of *α* and *β* ranged from 1.19 to 2.81 (*α*) and 0.0001 to 0.86 (*β*). Average values of *α* and *β* across the 11 species were 1.74 and 0.41, and thus similar to the values which here best approximated the canopy index. These results also indicate that species react differently to competition in terms of growth. We accounted for those differences with the parameters of our growth model, while keeping *α* and *β* constant.

While the correlation between the canopy index and the neighborhood competition index for individual trees was very similar in both census intervals (*r* = −0.84), the correlation for grid cells (recruitment) was higher in the first (*r* = −0.69) than in the second interval (*r* = −0.55). This might be a result of some extremely high values of the neighborhood competition index for grid cells in 1990 (Figure 2). In 1985, at the beginning of the first census interval, the BCI forest was still influenced by the consequences of an El Niño event in 1982 [49]. There were less tall trees and more tree fall gaps than in the 1990 census. Extremely high values of the neighborhood competition index can arise when there are many tall neighbors in the surroundings of a focal point, *i.e.* the center of a 5×5 m grid cell, or when one large tree is very close to the focal point. In 1985, such a constellation was less likely than in 1990 because of fewer large trees. This implies that estimating light with the neighborhood competition index for arbitrary points in the understorey should be applied with caution, especially when there are many tall trees.

A major drawback of the neighborhood competition indices compared to the canopy index is that they estimate the shade a tree casts, *i.e.* the geometry of its canopy, on the basis of its dbh, while the canopy index is based on direct observations of presence vs. absence of vegetation in six height layers. The allometry between dbh, height and canopy structure varies among species (*e.g.* [47]) and an approach that assumes allometry to be constant among an entire diverse community could lead to biased results. Another source of mismatch between neighborhood competition indices and the canopy index is that canopies are asymmetric and not centered above the stem base and that they might, for example, grow toward recent gaps in the canopy layer. Moreover, the canopy censuses, on which the canopy index relied, were performed every year during the two census intervals, and data were integrated over six years, whereas the neighborhood competition indices are based on one single census. This difference should also lower the correlation between the neighborhood competition index and the canopy index.

Since the regression coefficients of the transformation of neighborhood competition indices into the log(canopy index) varied only slightly between the first and second census interval, we propose that the coefficients of the general regression over both census intervals might be used to convert values of the neighborhood competition index into an approximation of light availability. However, the use of the optimal values of *α* and *β* identified in this study and the application of our transformation equations in forests with a significantly different structure should be treated with caution. The forest of BCI has an average basal area of 32 m^2^/ha (based on census data from 1985 and 1990), a canopy height of 33 m and 3 crown layers [45].

### Species' light response and between-species variation

Posterior means of the light response parameters *b_r_* and *b_g_* of the models with the neighborhood competition indices and the canopy index were highly correlated (*r*>0.85) for both processes, growth and recruitment, and for the interval-specific and the general transformed as well as for the raw untransformed neighborhood competition index. Thus, species rankings and between-species variation in response to light availability were described well by the neighborhood competition indices, indicating that even untransformed neighborhood competition indices can be used to determine species rankings when no canopy census data are available. Potentially, neighborhood competition indices that are transformed into the log(canopy index) could be used to determine the mode of light response according to the light response parameters *b_r_* and *b_g_*. However, classifications of species into light response groups were found to be rather inconsistent among the recruitment models for both census intervals. While transformed neighborhood competition index parameter estimates of *b_r_* were quite similar to those obtained by the canopy index in the first census interval, parameter estimates for the second census interval were underestimated by the neighborhood competition index, especially for high light responses. This deviation could be caused by some extremely high values of the neighborhood competition indices in the second census interval that led to a wider range of light estimates compared to the canopy index. This wider range of light estimates might have been compensated by smaller light response parameters as compared to canopy index models.

Analogously, for growth the values of the transformed neighborhood competition indices have a smaller range than the canopy index in the first census interval and constantly higher values for the light response parameter *b_g_*. The second census interval, in turn, revealed a different pattern that can not easily be explained by differences in light estimate distributions. However, based on the posterior mean of *b_g_*, almost all species were identified to show a decelerating response of growth to light, by the canopy index models as well as by the neighborhood competition index models. And the applicability of the neighborhood competition indices for assessing species rankings and between-species variation of light responses is not hampered by those deviations from the 1∶1 line, because the correlation is still very high.

### Limitations

There are several general problems inherent to such community-wide studies irrespective of the approach used to estimate light. Most important, both measures are surrogates of light availability, while leaves respond to light itself. As an example, the canopy index and the neighborhood competition index assume that crown transparency is the same for all species, although it has been shown that this is not the case [50].

Another caveat is that both growth and especially recruitment might respond to light availability over more than five years. For each census interval, we used data on light estimates that were, in the case of neighborhood competition indices, based on a single census and, in the case of the canopy index, integrated over six years. However, assuming that recruits have a radial growth of <1 mm/yr, they should on average be 10–20 years old when surpassing the 1 cm threshold. Thus, the light conditions in only a narrow time frame of a recruit's life could be captured, especially by the neighborhood competition index, and light conditions under which the seedling established and grew might have been different from those at the time of the census [51]. Therefore, it could be advantageous to include not only the last but also the penultimate census into the analysis to capture light environments from five and ten years before recruit data collection.

For the large number of rare species, predictions can only be made with a considerable amount of uncertainty. However, the hierarchical Bayesian approach explicitly accounts for that problem by superimposing a form of variation across the whole community in the parameter models [24], [25]. Moreover, the Bayesian approach allows calculating the credible intervals for parameters of every single species, so that uncertainty can be included in the analysis of model results.

### Conclusion

Using information about the three-dimensional canopy structure or neighborhood competition indices to estimate the species-specific strength of light response in general yielded slightly different results. However, given that the ranking of species regarding their light response is remarkably stable, neighborhood competition indices open the possibility to produce rankings and estimates of between-species variation in light response for a large number of forest plots where no canopy census data are available but where dbh and position of trees are recorded. We believe that the gain in information by applying this straightforward method to many more forests largely outweighs the imprecision. Comparative studies might shed light on the nature of light response on a global scale and reveal possible relationships between light response and species' characteristics like functional traits, abundance, or phylogenetic relationship, or plot characteristics, such as soil, climate, or species richness.

## Supporting Information

Appendix S1 The R code for the hierarchical Bayesian model for recruitment.
(TXT)

Appendix S2 The code for the hierarchical Bayesian growth model in C++ to be run with Filzbach.
(CPP)

Appendix S3 Light response parameter estimates from recruitment and growth models with the untransformed neighborhood competition index compared to models with the canopy index.
(PDF)

## References

[1] Grubb PJ (1977) The maintenance of species-richness in plant communities: the importance of the regeneration niche. Biological reviews of the Cambridge Philosophical Society 52 (1): 107–145.

[2] Ricklefs RE (1977) Environmental heterogeneity and plant species diversity: a hypothesis. American Naturalist 111(978): 376–381.

[3] Denslow JS, Schultz JC, Vitousek PM, Strain BR (1990) Growth Responses of Tropical Shrubs to Treefall Gap Environments. Ecology 71(1): 165–179.

[4] King DA, Davies SJ, Supardi MN, Tan S (2005) Tree growth is related to light interception and wood density in two mixed dipterocarp forests of Malaysia. Functional Ecology 19(3): 445–453.

[5] Hartshorn GS (1980) Neotropical forest dynamics. Biotropica 12 (S): : 23–30.

[6] Welden CW, Hewett SW, Hubbell SP, Foster RB (1991) Sapling survival, growth, and recruitment: relationship to canopy height in a neotropical forest. Ecology 72(1): 35–50.

[7] Chesson PL (1985) Coexistence of competitors in spatially and temporally varying environments: A look at the combined effects of different sorts of variability. Theoretical Population Biology 28(3): 263–287.

[8] Sack L, Grubb PJ (2001) Why do species of woody seedlings change rank in relative growth rate between low and high irradiance. Functional Ecology 15(2): 145–154.

[9] Agyeman VK, Swaine MD, Thompson J (1999) Responses of tropical forest tree seedlings to irradiance and the derivation of a light response index. Journal of Ecology 87(5): 815–827.

[10] Kitajima K (1994) Relative importance of photosynthetic traits and allocation patterns as correlates of seedling shade tolerance of 13 tropical trees. Oecologia 98(3–4): 419–428. 28313920 10.1007/BF00324232

[11] Poorter L (1999) Growth responses of 15 rain-forest tree species to a light gradient: the relative importance of morphological and physiological traits. Functional Ecology 13(3): 396–410.

[12] Gilbert B, Wright SJ, Muller-Landau HC, Kitajima K, Hernandez A (2006) Life history trade-offs in tropical trees and lianas. Ecology 87(5): 1281–1288. 16761606 10.1890/0012-9658(2006)87[1281:lhtitt]2.0.co;2

[13] Rüger N, Huth A, Hubbell SP, Condit R (2009) Response of recruitment to light availability across a tropical lowland rain forest community. Journal of Ecology 97(6): 1360–1368.

[14] Rüger N, Berger U, Hubbell SP, Vieilledent G, Condit R (2011) Growth Strategies of Tropical Tree Species: Disentangling Light and Size Effects. PLoS ONE 6(9).. 10.1371/journal.pone.0025330 PMC3178650 21966498

[15] Peacock J, Baker TR, Lewis SL, Lopez-Gonzalez G, Phillips OL (2007) The RAINFOR database: monitoring forest biomass and dynamics. Journal of Vegetation Science 18(4): 535–542.

[16] Condit R, Hubbell SP, Foster RB (1996) Assessing the response of plant functional types to climatic change in tropical forests. Journal of Vegetation Science 7(3): 405–416.

[17] Wright SJ, Muller-Landau HC, Condit R, Hubbell SP (2003) Gap-dependent recruitment, realized vital rates, and size distributions of tropical trees. Ecology 84(12): 3174–3185.

[18] Condit R (1998) Tropical forest census plots. Methods and results from Barro Colorado Island, Panama and a comparison with other plots. New York:Springer .211 S p .

[19] Brown S, Gillespie AJ, Lugo AE (1989) Biomass estimation methods for tropical forests with applications to forest inventory data. Forest Science 35 (4): 881–902.

[20] Lev-Yadun S (2005) Shade avoidance and Zahavi's handicap principle in dense plant populations. Biological Journal of the Linnean Society 84(2): : 313-319.

[21] Hegyi F (1974) A simulation model for managing jack-pine stands. In: Royal College of Forestry, editor. Growth models for tree and stand simulation. Stockholm, Sweden. pp. 74–90.

[22] Martin GL, Ek AR (1984) A comparison of competition measures and growth-models for predicting plantation red pine diameter and height growth. Forest Science 30(3): 731–743.

[23] Tomé M, Burkhart HE (1989) Distance-dependent competition measures for predicting growth of individual trees. Forest Science 35(3): 816–831.

[24] Clark JS (2005) Why environmental scientists are becoming Bayesians. Ecology Letters 8(1): 2–14.

[25] Condit R, Ashton P, Bunyavejchewin S, Dattaraja HS, Davies S, et al (2006) The importance of demographic niches to tree diversity. Science 313(5783): 98–101. 16763113 10.1126/science.1124712

[26] Leigh EG, Rand AS, Windsor DM (1996) The ecology of a tropical forest. .Washington (D.C): Smithsonian Institution. (1 vol: . 503 p.

[27] Leigh EG (1999) Tropical forest ecology. A view from Barro Colorado Island. New York: Oxford University Press. xvi, 245 p.

[28] Windsor DM (1990) Climate and Moisture Variability in a Tropical Forest: Long-term Records from Barro Colorado Island, Panamá. Smithsonian Contributions to the Earth Sciences 29..

[29] Das A (2012) The effect of size and competition on tree growth rate in old-growth coniferous forests. Canadian Journal of Forest Research 42(11): 1983–1995.

[30] Rüger N, Huth A, Hubbell SP, Condit R (2011) Determinants of mortality across a tropical lowland rainforest community. Oikos 120(7): 1047–1056.

[31] Wirth R, Weber B, Ryel RJ (2001) Spatial and temporal variability of canopy structure in a tropical moist forest. Acta Oecologica-International Journal of Ecology 22(5–6): 235–244.

[32] Wykoff W, Crookston N, Stage A (1982) User's guide to the Stand Prognosis Model: U.S. Dept. of Agriculture, Forest Service, Intermountain Forest and Range Experiment Station .

[33] Canham CD, LePage PT, Coates KD (2004) A neighborhood analysis of canopy tree competition: effects of shading versus crowding. Canadian Journal of Forest Research 34(4): 778–787.

[34] Peters HA (2003) Neighbour-regulated mortality: the influence of positive and negative density dependence on tree populations in species-rich tropical forests. Ecology Letters 6(8): 757–765.

[35] Stoll P, Newbery DM (2005) Evidence of species-specific neighborhood effects in the dipterocarpaceae of a Bornean rain forest. Ecology 86(11): 3048–3062. 10.1890/13-0366.1 24597229

[36] Comita LS, Muller-Landau HC, Aguilar S, Hubbell SP (2010) Asymmetric Density Dependence Shapes Species Abundances in a Tropical Tree Community. Science 329(5989): 330–332. 20576853 10.1126/science.1190772

[37] Gourlet-Fleury S, Houllier F (2000) Modelling diameter increment in a lowland evergreen rain forest in French Guiana. Forest Ecology and Management 131(1–3): 269–289.

[38] Uriarte M, Canham CD, Thompson J, Zimmerman JK, Brokaw N (2005) Seedling recruitment in a hurricane-driven tropical forest: light limitation, density-dependence and the spatial distribution of parent trees. Journal of Ecology 93(2): 291–304.

[39] Comita LS, Condit R, Hubbell SP (2007) Developmental changes in habitat associations of tropical trees. Journal of Ecology 95(3): 482–492.

[40] Muller-Landau HC, Wright SJ, Calderon O, Condit R, Hubbell SP (2008) Interspecific variation in primary seed dispersal in a tropical forest. Journal of Ecology 96(4): 653–667.

[41] John R, Dalling JW, Harms KE, Yavitt JB, Stallard RF, et al (2007) Soil nutrients influence spatial distributions of tropical tree species. Proceedings of the National Academy of Sciences of the United States of America 104(3): 864–869. 17215353 10.1073/pnas.0604666104 PMC1783405

[42] Bolker BM (2008) Ecological models and data in R. Princeton, N.J: Princeton University Press. vii, 396 p.

[43] Gelman A (2004) Bayesian data analysis. .London, [etc]: Chapman & Hall. XXV, 668 p.

[44] Gelman A, Rubin DB (1992) Inference from iterative simulation using multiple sequences. Statistical Science 7: 457–511.

[45] R Core Team (2012) R: A Language and Environment for Statistical Computing. Vienna, Austria.

[46] Chave J, Condit R, Aguilar S, Hernandez A, Lao S, et al (2004) Error propagation and scaling for tropical forest biomass estimates. Philosophical Transactions of the Royal Society of London Series B-Biological Sciences 359(1443): 409–420. 10.1098/rstb.2003.1425 PMC1693335 15212093

[47] Bohlman S, O′Brien S (2006) Allometry, adult stature and regeneration requirement of 65 tree species on Barro Colorado Island, Panama. Journal of Tropical Ecology 22 (Part2): 123–136.

[48] Uriarte M, Canham CD, Thompson J, Zimmerman JK (2004) A neighborhood analysis of tree growth and survival in a hurricane-driven tropical forest. Ecological Monographs 74(4): 591–614.

[49] Condit R, Hubbell SP, Foster RB (1996) Changes in tree species abundance in a Neotropical forest: Impact of climate change. Journal of Tropical Ecology 12 (Part 2): 231–256.

[50] Canham CD, Finzi AC, Pacala SW, Burbank DH (1994) Causes and consequences of resource heterogeneity in forests - interspecific variation in light transmission by canopy trees. Canadian Journal of Forest Research 24(2): 337–349.

[51] Kobe RK (1999) Light gradient partitioning among tropical tree species through differential seedling mortality and growth. Ecology 80(1): 187–201.

