# Supplementary material for: Response of Demographic Rates of Tropical Trees to Light Availability: Can Position-Based Competition Indices Replace Information from Canopy Census Data?
Source: PLoS One. 2013 Dec 6;8(12):e81787. doi: 10.1371/journal.pone.0081787 (PMC3855810; doi:10.1371/journal.pone.0081787)
Supplement: Appendix S3 — Light response parameter estimates from recruitment and growth models with the untransformed neighborhood competition index compared to models with the canopy index. (PDF) [file pone.0081787.s003.pdf]

Appendix S3

Light response parameter estimates from recruitment and growth models with the untransformed neighborhood competition index compared to models with the canopy index.

The figures below show species-specific posterior means of the light response parameter b_r for recruitment models (Figure S1) and b_g for growth models (Figure S2). Parameter estimates from models with either the canopy index or the untransformed neighborhood competition index as light estimate are compared. Since the untransformed neighborhood competition index values were very large, we divided them arbitrarily by -5000 (recruitment) or by -500 (growth). This way the models converged faster. By taking the negative value, we converted the neighborhood competition index to represent light rather than shade.

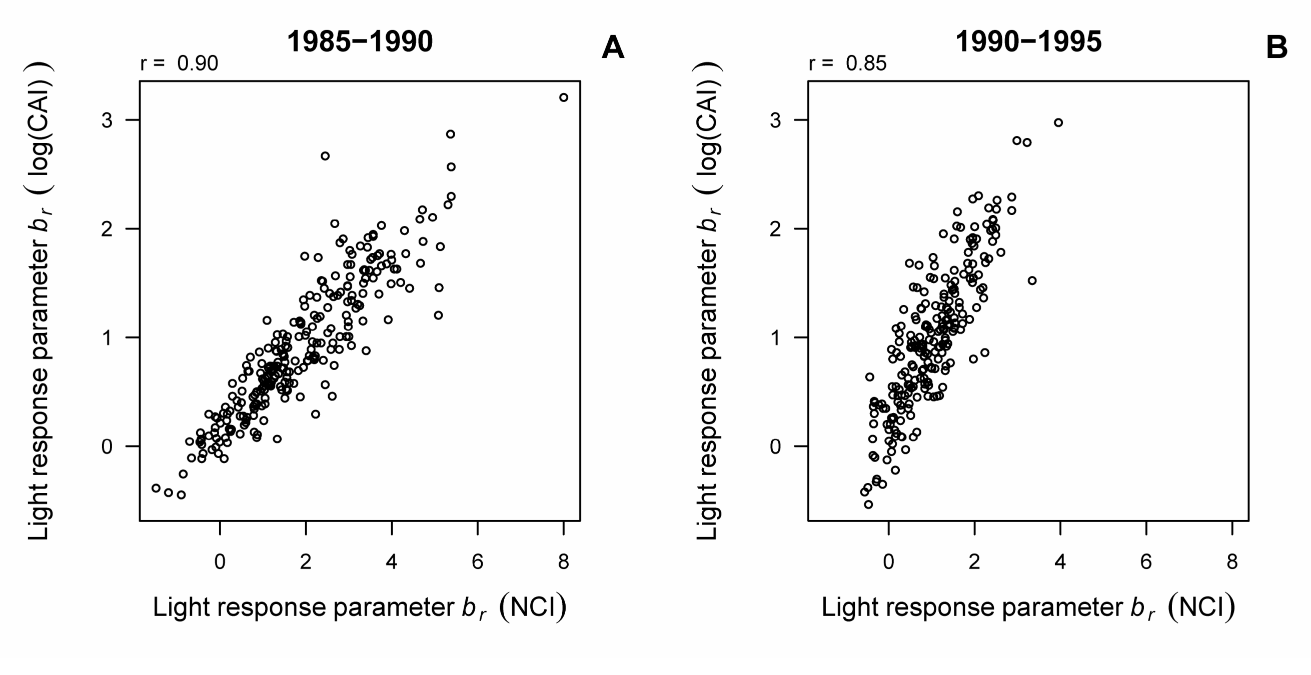

Figure S1: Species-specific light response of recruitment (b_r) in models with neighborhood competition index versus canopy index. Panels show posterior means of b_r in models with the untransformed neighborhood competition index (NCI) compared to b_r in models with the canopy index (log(CAI)) for the first (A) and the second census interval (B). The correlation between the coefficients (*r*) is indicated.

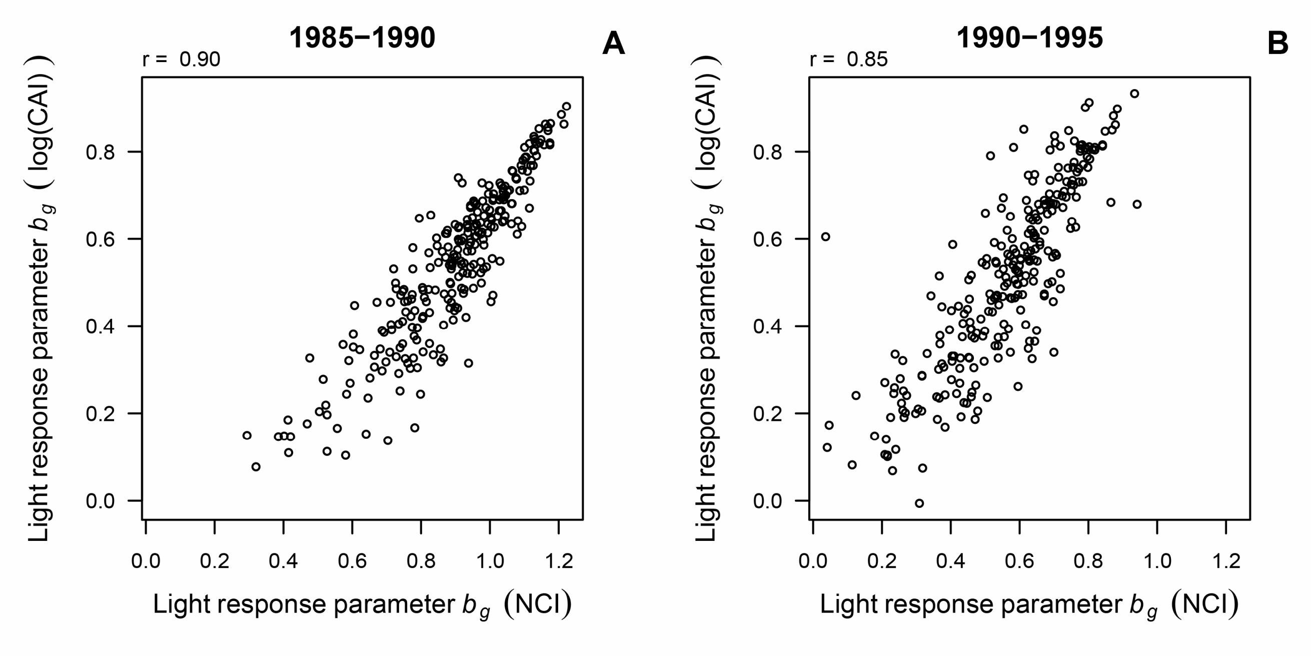

Figure S2: Species-specific light response of growth (b_g) in models with neighborhood competition index versus canopy index. Panels show posterior means of b_g in models with the untransformed neighborhood competition index (NCI) compared to b_g in models with the canopy index (log(CAI)) for the first (A) and the second census interval (B). The correlation between the coefficients (*r*) is indicated.
